# Supplementary material for: Neutralizing antibodies against SARS-CoV-2 variants of concern elicited by the comirnaty COVID-19 vaccine in nursing home residents
Source: Sci Rep. 2022 Mar 8;12:3788. doi: 10.1038/s41598-022-07849-2 (PMC8904770; doi:10.1038/s41598-022-07849-2)
Supplement: Supplementary file 1 — Supplementary Information 1. [file 41598_2022_7849_MOESM1_ESM.docx]

| **Supplementary Table 1. Correlation between SARS-CoV-2 neutralizing antibodies and anti-RBD antibodies in fully vaccinated nursing home residents and healthy controls.** | | | | | | |  |
| --- | --- | --- | --- | --- | --- | --- | --- |
|  | | | | | | | |
| SARS-CoV-2 variant | Group 1^a^ | | | | Group 2^b^ | | |
|  | SARS-CoV-2 naïve | | SARS-CoV-2 experienced | |  |  |  |
|  | Rho value^c^ | *P value* | Rho value^c^ | *P value* | Rho value^c^ | *P value* | |
| Beta | 0.81 | <0.001 | 0.91 | 0.005 | 0.81 | <0.001 | |
| Gamma | 0.90 | <0.001 | 0.86 | 0.011 | 0.83 | <0.001 | |
| Delta | 0.75 | <0.001 | 0.79 | 0.036 | 0.79 | <0.001 | |
| Epsilon | 0.79 | <0.001 | 0.69 | 0.069 | 0.85 | <0.001 | |
| Wuhan-Hu-1 | 0.83 | <0.001 | 0.52 | 0.197 | 0.88 | <0.001 | |
| ^a^Nursing home residents and ^b^Healthy individuals fully vaccinated with the Comirnaty vaccine.  ^c^Spearman Rank test | | | | | | | |
